# Insight into the role of *Streptococcus suis* zinc metalloprotease C from the new serotype causing meningitis in piglets

**DOI:** 10.1186/s12917-024-03893-4

**Published:** 2024-07-30

**Authors:** Qibing Gu, Peijuan He, Qiankun Bai, Xiaojun Zhong, Yue Zhang, Jiale Ma, Huochun Yao, Zihao Pan

**Affiliations:** 1 College of Veterinary Medicine, Nanjing Agricultural University, Nanjing, 210095 China; 2 Key Lab of Animal Bacteriology, Ministry of Agriculture, Nanjing, 210095 China; 3 OIE Reference Lab for Swine Streptococcosis, Nanjing, 210095 China; 4 Present Address: Master Shanxi Animal Health and Slaughtering Management Station, Xian, Shanxi Province 710016 China; 5 Present Address: College of Animal Science and Technology, College of Veterinary Medicine, Zhejiang A & F University, Hangzhou, 311300 China; 6 Present Address: College of Veterinary Medicine, Henan Agricultural University, Zhengzhou, 450046 P. R. China

**Keywords:** Zinc metalloprotease, Virulence, Serotype Chz, *Streptococcus suis*, Meningitis, Protective antigen

## Abstract

*Streptococcus suis* (*S. suis*) is an important gram-positive pathogen and an emerging zoonotic pathogen that causes meningitis in swine and humans. Although several virulence factors have been characterized in *S. suis*, the underlying mechanisms of pathogenesis are not fully understood. In this study, we identified Zinc metalloproteinase C (ZmpC) probably as a critical virulence factor widely distributed in *S. suis* strains. ZmpC was identified as a critical facilitator in the development of bacterial meningitis, as evidenced by the detection of increased expression of TNF-α, IL-8, and matrix metalloprotease 9 (MMP-9). Subcellular localization analysis further revealed that ZmpC was localized to the cell wall surface and gelatin zymography analysis showed that ZmpC could cleave human MMP-9. Mice challenge demonstrated that ZmpC provided protection against *S. suis* CZ130302 (serotype Chz) and ZY05719 (serotype 2) infection. In conclusion, these results reveal that ZmpC plays an important role in promoting CZ130302 to cause mouse meningitis and may be a potential candidate for a *S. suis* CZ130302 vaccine.

**Supplementary Information:**

The online version contains supplementary material available at 10.1186/s12917-024-03893-4.

## Introduction

*Streptococcus suis* (*S. suis*) is a major pathogen causing swine streptococcus infection and is an emerging zoonotic pathogen [1]. *S. suis* causes a wide range of diseases, including septicemia, meningitis, pneumonia, and arthritis [2]. *S. suis* isolates are classified into 33 serotypes based on capsular polysaccharides and to date, 28 novel *cps* types (NCL1- NCL27 and Chz) *S. suis* strains have been identified [3–7]. Although several virulence factors, such as extracellular protein factor (EPF), muramidase-related protein (MRP), and suilysin (SLY), have been identified in *S. suis*, the Chz type strain CZ130302, which causes acute meningitis, lacks these virulence markers [8, 9]. The pathogenic molecular mechanism of CZ130302 requires further exploration.

Zinc metalloproteases (Zmps) are a widely distributed and diverse family of proteolytic enzymes with a conserved HE*XX*H…E motif [10]. An increasing body of evidence demonstrates that Zmp plays important roles in the pathogenic processes of bacteria. The lethal toxin, the major virulence factor of *Bacillus anthracis*, contains the effector moiety lethal factor, which acts as a Zmp specific target protein kinase, thereby contributing to the anthrax [11]. In *Listeria monocytogenes*, Mp1 is a Zmp that activates the virulence factor phospholipase C [12]. It is well known that IgA, as an immunoglobulin, plays an important role in the elimination of pathogens from the host. To colonize the host, bacteria have evolved mechanisms to resist the host’s innate immunity by expressing IgA proteases [13]. The IgA protease is a Zmp that cleaves human IgA and has been characterized in various bacterial species [14–16]. There are a large number of Zmps on the cell surface of *Streptococcus pneumoniae*, and these Zmps are classified into four different groups: ZmpA (IgA protease), ZmpB, ZmpC, and ZmpD [17]. The N-terminal region of these Zmps contains the LPXTG motif, which is important for the localization of Zmps. The C-terminal part of Zmps comprises a proteolytic domain and contains motifs characteristic of zinc metalloproteinases. Many Zmps have been identified as critical for the virulence of *S. pneumoniae* [17]. Phylogenetic analysis has confirmed that the Zmp of *S. suis* is a homologue of *S. pneumoniae* ZmpC [17]. These studies propose that Zmp in *S. suis* might play an important role in the pathogenic process and deserves further exploration.

It has been demonstrated that *S. pneumoniae* ZmpC can activate human matrix metalloproteinase 9 (MMP-9) [18]. MMP-9 is a zinc-dependent matrix-degrading enzyme that can disrupt the blood-brain barrier (BBB) [19]. High levels of MMPs are present in the cerebrospinal fluid of patients with bacterial meningitis [20]. In a rat model of meningitis, the transcriptional level of MMP-9 in brain tissue was significantly increased [21]. These studies suggest that the activation of MMP-9 plays a critical role in the development of meningitis. Can Zmp in *S. suis* serotype Chz also activate MMP-9? What is the role of Zmp in the meningitis-caused strain CZ130302 in the progression of meningitis? The exploration of these questions will help to understand the molecular mechanism of *S. suis* meningitis.

In the present study, we analyzed the distribution of Zmp in *S. suis* and identified the role of ZmpC in the virulence of serotype Chz strain CZ130302. Further research has found that ZmpC plays a significant role in the meningitis induced by CZ130302. ZmpC, located on the bacterial surface, induces the host to produce a high antibody titer against lethal infection caused by *S. suis* CZ130302. Understanding the role of Zmp will provide new insights for the study of the pathogenic mechanism of *S. suis* CZ130302.

## Materials and methods

### Ethics statement

Five-week-old female specific pathogen free (SPF) BALB/c mice were purchased from the Comparative Medicine Center of Yangzhou University. All animal experiments were performed in strict accordance with the animal welfare standards of the Guidelines of the Jiangsu Provincial Animal Research Committee (License Number: SYXK (SU) 2017–0007), and were approved by the Animal Ethics Committee of Nanjing Agricultural University.

### Bacterial strains, plasmids, and culture conditions

Chz type strain CZ130302 was isolated from the brain tissue of piglets with acute meningitis [4]. The strains and plasmids used in this study are shown in Table 1. All *S. suis* strains were cultivated in Todd-Hewitt broth (THB, BD, USA) containing 3% fetal bovine serum, or on solid medium containing 5% sheep blood (v/v), and were cultured at 37 °C in an incubator with 5% CO2. To screen for mutants, 100 µg/mL spectinomycin (Spc, Sigma-Aldrich, USA) or 10% sucrose (w/v) was added to the medium as needed. *Escherichia coli* strains were grown in Luria-Bertani (LB, BD, USA) medium at 37 °C. 50 µg/mL kanamycin (Kan, Sigma-Aldrich, USA) was added to LB medium to construct recombinant plasmids when necessary.

**Table 1:** Bacterial strains and plasmids used in this study

Bacterial strains/Plasmids	Description^a^	Reference
**Strains**		
CZ130302	Serotype Chz *S. suis* strain	Collected in our lab
Δ*zmpC*	Deletion mutant of zmpC with CZ130302 background	This study
Δ*zmpE*	Deletion mutant of zmpE with CZ130302 background	This study
Δ*zmpN*	Deletion mutant of zmpN with CZ130302 background	This study
Δ*zmpB*	Deletion mutant of zmpB with CZ130302 background	This study
ZY05719	Serotype 2 *S. suis* strain	Collected in our lab
DH5α	Cloning host for maintaining the recombinant plasmids	Invitrogen, USA
BL21 (DE3)	Host for expressing proteins	Invitrogen, USA
**Plasmids**		
Pet28a(+)	Expression vector, Kan^r^	Invitrogen, USA
Pet28a-ZmpC-M26	Pet-28a containing zmpC-M26 gene, Kan^r^	This study
Pet28a-ZmpE-M26	Pet-28a containing zmpE-M26 gene, Kan^r^	This study

^a^kanamycin resistant

### Bioinformatics

The Zmp of *S. suis* in the NCBI database (https://www.ncbi.nlm.nih.gov/) was searched and analyzed according to the conserved HE*XX*H…E motif. The data was visualized by GraphPad Prism 8. IgA1 protease, ZmpC, ZmpD, and ZmpB in *S. pneumoniae* were used as references, and a phylogenetic tree of *S. suis* Zmp was constructed by Neighbor-Joining Tree using MEGA-X software. The three-dimensional structures of ZmpC in *S. suis* CZ130302 and *S. pneumoniae* TIGR4 were predicted by the online prediction website SWISS-MODEL (https://swissmodel.expasy.org).

### Construction of the Zmp gene deletion mutants

Zmp mutants were constructed via natural DNA transformation with some modifications [22]. The sequences of all primers used to construct deletion strains are listed in Supplementary material 1. The upstream and downstream fragments of Zmp were amplified by polymerase chain reaction (PCR) with primer pairs from the genomic DNA of CZ130302. The upstream and downstream fragments were fused with the sacB-spc cassette through overlap PCR. The fusion fragment and synthetic peptide were added to the 100 µl bacterial suspension (OD_600_ ≈ 0.042). Samples were incubated at 37 °C for 2 h under static conditions and then plated on THB containing Spc. The positive mutations carrying sacB-spc were then detected by PCR with primers. The *sacB* gene is sensitive to sucrose and can be used as a negative control. The fusion fragment without the cassette was transformed into the primary mutant to obtain the positive clone that did not carry the resistance gene.

### Growth curve and CFU determination

The CZ130302, Δ*zmpC*, Δ*zmpE*, Δ*zmpN* and Δ*zmpB* strains in log phase were diluted 1:100 in fresh THB broth. The strains were cultured in a shaker at 37 °C, and the OD_600_ value was measured and recorded every 1 h. In addition, samples were diluted with sterile PBS every 2 h. Serial 10-fold dilutions of the bacterial suspensions were plated on THB agar and then incubated for 24 h at 37 °C, after which the colony forming units (CFU) were counted. The experiment was repeated independently three times. The growth curves and CFU assay were drawn using GraphPad Prism 8 software.

### Mouse infection tests

To assess the virulence of the Δ*zmp* strains, BALB/c mice were randomly divided into 5 groups of 10 mice each and challenged with the strains at a dose of 5 × 10^7^ colony forming units (CFU)/mouse by intraperitoneal injection. Another group of 10 mice was challenged with PBS as a control group. The clinical symptoms (neurological symptoms: lethargy, coma, circling, and convulsions) and survival of the mice were continuously monitored for 7 days. In addition, bacterial load analysis was used to assess the proliferation capacity of Δ*zmpC* in mice. Two groups of 10 mice each were challenged with the indicated *S. suis* CZ130302 or Δ*zmpC* at a dose of 1 × 10^8^ CFU/mouse through intraperitoneal injection, respectively. Subsequently, the brain, liver, and spleen were harvested, weighed, and homogenized in phosphate buffer solution (PBS) at 12 h post-infection. The infected mice were anaesthetised with 3% isoflurane inhalation and euthanised with CO_2_ at 12 h after infection according to the reference [23]. Bacteria were isolated by plating serial 10-fold dilutions on a THB agar (THA) medium to enumerate CFU.

To determine the protective potential of recombinant protein in BALB/c mice, mice immunized with recombinant protein ZmpC-M26 or adjuvant ISA201 (control) (Seppic, France) were challenged with CZ130302 (Chz type) and ZY07519 (serotype 2), respectively. Mice were randomly divided into 4 groups, and 8 mice in each group were immunized 24 days and then challenged with CZ130302 at a dose of 2 × 10^7^ CFU/mouse or ZY05719 at a dose of 2 × 10^8^ CFU/mouse. Finally, the clinical symptoms (neurological symptoms: lethargy, coma, circling, and convulsions) and survival of the mice were continuously observed and recorded.

### RNA isolation, RT-PCR, and qRT-PCR

Total RNA from bacteria in log phase and the infected host brain tissue cells was extracted using TRIzol (Vazyme, Nanjing, China) according to the manufacturer’s instructions. After removing contaminating DNA with DNase I (Vazyme, Nanjing, China), RNA was used as a template to synthesize cDNA using the PrimeScript RT reagent kit (Vazyme, Nanjing, China). The QuantStudio 6 Flex RT-PCR System and ChamQ Universal SYBR qPCR Master Mix (Vazyme, Nanjing, China) were used to determine the transcription of selected genes. Transcript of β-Actin acts as the control to normalize the relative amount of host cell target gene mRNA [24]. The relative amount of *S. suis* target gene mRNA was normalized to the gene *gapdh* [25].

### Purification of protein and preparation of polyclonal antibody

The sequence containing the M26 domain was amplified from the CZ130302 genomic DNA. The sequences were digested and ligated into pET-28a plasmid and the recombinant plasmids were transferred into BL21 (DE3). The recombinant proteins were purified by Ni-NTA Spin Columns (QIAGEN, Germany) from BL21 (DE3) carrying the recombinant pET-28a plasmid after isopropyl*-*β*-*d*-*thiogalactopyranoside (IPTG) (Thermo Fisher Scientific, USA) induction (0.1 mM) for 16 h at 16° C. Protein concentrations were determined by BCA (Thermo Fisher Scientific, USA) protein assays. BALB/C mice in each group were immunized via multipoint intradermal injection for three times at 14 days intervals with 100 µg recombinant proteins. The polyclonal antiserum was collected from immunized mice after the third immunization, and titers were determined by enzyme-linked immunosorbent assay (ELISA).

### Gelatin zymographic analysis

Gelatin zymography assay was performed according to the literature with some modifications [26]. Briefly, the purified ZmpC-M26 (900 ng to 1300 ng) and ZmpE-M26 (900 ng to 1300 ng) were incubated with human MMP-9 (Shanghai You Ning Wei Biotechnology Co. Ltd, Shanghai, China) in a buffer containing zinc ions for 1 h at 37 °C, respectively. The samples were separated on SDS-PAGE (1% gelatin, Invitrogen, USA) at 4 °C. The protein gel was then washed four times with the eluent (2.5% Triton-X, 50 mmol/L Tris-HCl, 5 mmol/L CaCl_2_, 1µmol/L ZnCl_2_, pH 7.6) at 4 °C for 1 h each time. The protein gel was washed twice with rinse solution (50 mmol/L Tris-HCl, 5 mmol/L CaCl_2_, 1µmol/L ZnCl_2_, pH 7.6) at 4 °C for 40 min each. The protein gel was then placed in the incubation solution (50 mmol/L Tris-HCL, 5 mmol/ CaCl_2_, 1 µmol/L ZnCl_2_, 0.02% Brij-35) and incubated at 37 °C for 48–72 h. Finally, the protein gel was stained overnight in staining solution (0.05% Coomassie brilliant blue R-250, 30% methanol, 10% acetic acid). Images were collected after the protein gel was destained.

### Western blot analysis

The extraction of cell wall proteins was with reference to the literature with appropriate modifications [27]. Briefly, 30 mL of bacteria in log phase were collected and washed three times with sterile PBS. The precipitates were then resuspended in 1.2 mL of sample preparation solution (125 U/ml mutanolysin (Sigma-Aldrich, USA), 25% sucrose, 30 mM Tris-HCl (pH 7.5), 3 mM MgCl2) and incubated at 37 °C for 1 h. After incubation, the cell lysates were centrifuged at 8000 rpm for 10 min at 4 °C. 120 µL of cooled TCA (trichloroacetic acid, Sinopharm Chuan Kang Pharmaceutical Co., Ltd. China) at a final concentration of 10% was added to the supernatant, and the mixtures were incubated for 30 min in ice-water. The mixtures were then centrifuged at 8000 rpm for 10 min at 4 °C. The protein precipitate was washed twice with chilled acetone (Sinopharm Chuan Kang Pharmaceutical Co., Ltd. China) and allowed to air dry. The cell wall proteins were separated by SDS-PAGE, and then transferred to polyvinylidene fluoride (PVDF) membranes (Millipore, USA) and blocked with 5% (w/v) skimmed milk for 2 h at 37 °C. Subsequently, the membrane was washed three times with PBST, incubated with the prepared polyclonal antibodies (anti-ZmpC-M26) at 37° C, diluted 1:1000 for 2 h. After three washes, the processed membranes were incubated with HRP-conjugated secondary antibody (1:8000) at 37 °C for 45 min. Positive bands were detected with ECL kit (Vazyme, Nanjing, China).

### Indirect immunofluorescence analysis

The bacteria cultured to the log phase were washed with 1×PBS, and then 5 µl of bacteria were taken on a coverslips and fixed in 4% paraformaldehyde. Bacterial samples were blocked with 5% BSA and labeled with antiserum (anti-ZmpC-M26 or negative serum) diluted 1:200 at 37 °C for 2 h. After thrice washed, the bacteria samples were incubated with secondary antibody, Alexa Fluor 488- conjugated (Thermo Fisher, USA), diluted at 1:400. After incubation at 25 °C for 2 h, the samples were washed 3 times with 1×PBS and stained with DAPI (4′, 6-diamidino-2-phenylindole) (KeyGEN BioTECH, Nanjing, China) for 5 min. Finally, the treated samples were observed on a laser scanning confocal microscope (Leica Sp5 AOBS confocal system, Leica, Germany).

### Statistical analysis

All experiments were repeated at least three times. Data were assessed for normality using the Shapiro-Wilk test. Statistical analyses were performed using GraphPad Prism version 8. The survival rate of mice was analyzed using the Log-rank (Mantel-Cox) test. Statistical significance was set at a *P* value of <0.05, and an unpaired two-tailed Student’s t-test was applied to analyze the data.

## Results

### Distribution and evolutionary analysis of Zmp in *S. suis*

**Fig. 1:**
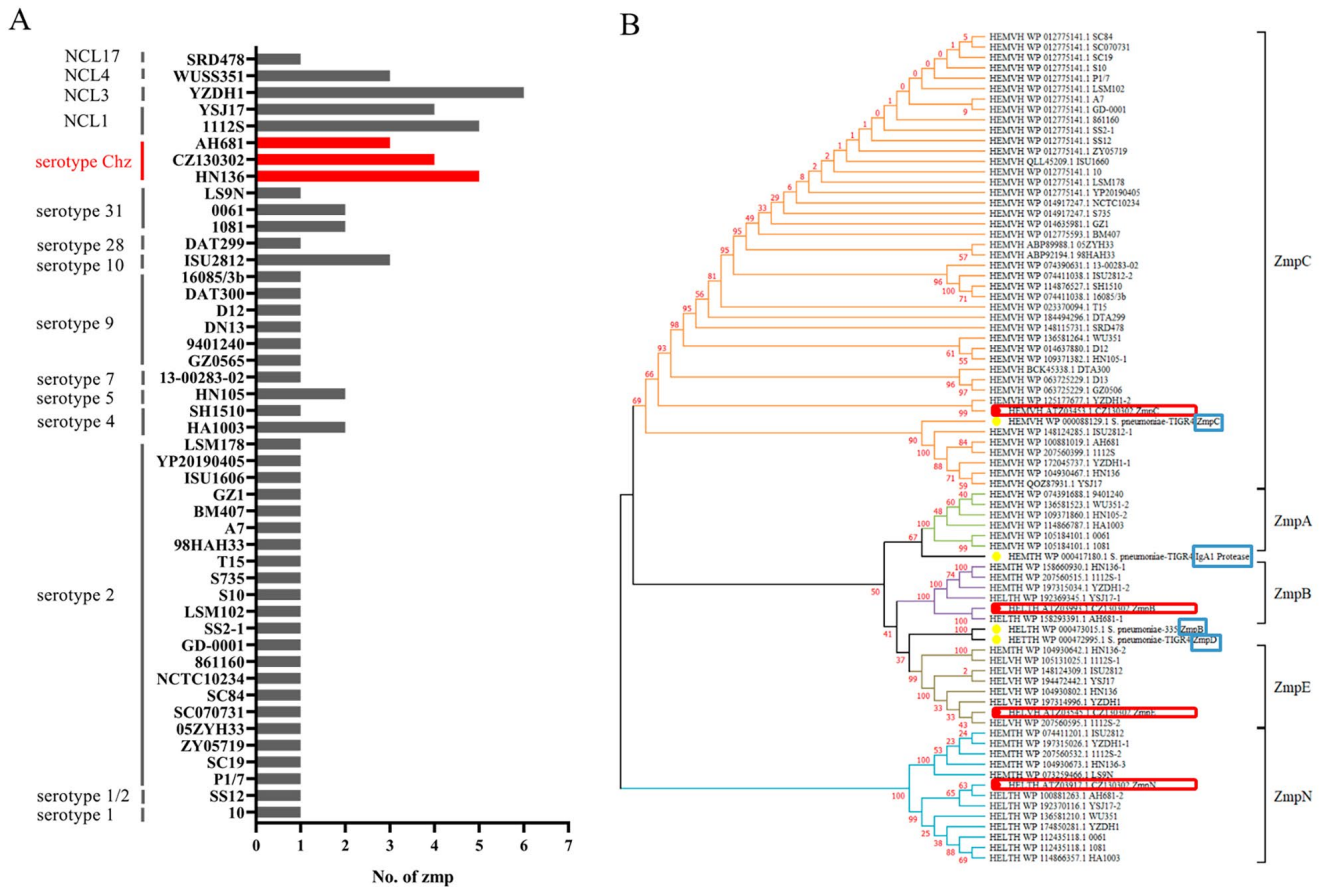
Distribution and phylogenetic analysis of Zmp in *S. suis*. (**A**) The NCBI database was searched for Zmp of *S. suis* using conserved HE*XX*H…E motifs. The number of Zmps in each *S. suis* strain was analyzed using GraphPad Prism 8. Chz type strains are highlighted in red. (**B**) Phylogenetic analysis was constructed by Neighbor-Joining Tree using MEGA-X software. IgA1 proteases (H020_RS0105710), ZmpC (H020_RS0100375), ZmpD (H020_RS0103275) and ZmpB (C4N11_03120) in *S. pneumoniae* were used as references and are highlighted with blue boxes. Zmps in the Chz type CZ130302 strain are highlighted with red boxes

Although Zmps are widely existent in *S. pneumoniae* and have been characterized [17], there are few studies on Zmp in *S. suis*. Therefore, we analyzed the distribution of Zmp in *S. suis* from the NCBI database based on the conserved HE*XX*H…E motif. The result showed that Zmp was distributed in different serotypes of *S. suis* (Fig. 1A). Furthermore, Zmps were more present in the novel *cps* types (Chz, NCL1, NCL3, NCL4 and NCL17) *S. suis* strains, suggesting a different role for Zmp in the novel *cps* types *S. suis* compared to common serotypes (e.g. serotype 2). Further phylogenetic analysis found that Zmps in *S. suis* were classified into five distinct groups: ZmpA, ZmpB, ZmpC, ZmpE, and ZmpN (Fig. 1B). The ZmpN cluster was distantly related to other four clusters, indicating that ZmpN is a new class of zinc metalloproteinases, which suggests that ZmpN may have different biological functions. And phylogenetic analysis also showed that ZmpC is widespread in *S. suis*, suggesting that ZmpC may play an important role in *S. suis*. Further analysis found that the Chz type strain CZ130302 contained four different Zmps: ZmpB, ZmpC, ZmpE, and ZmpN (Fig. 1B). These results reveal the biological functional importance of Zmp in CZ130302.

### ZmpC contributes to the virulence of chz type strain CZ130302

**Fig. 2:**
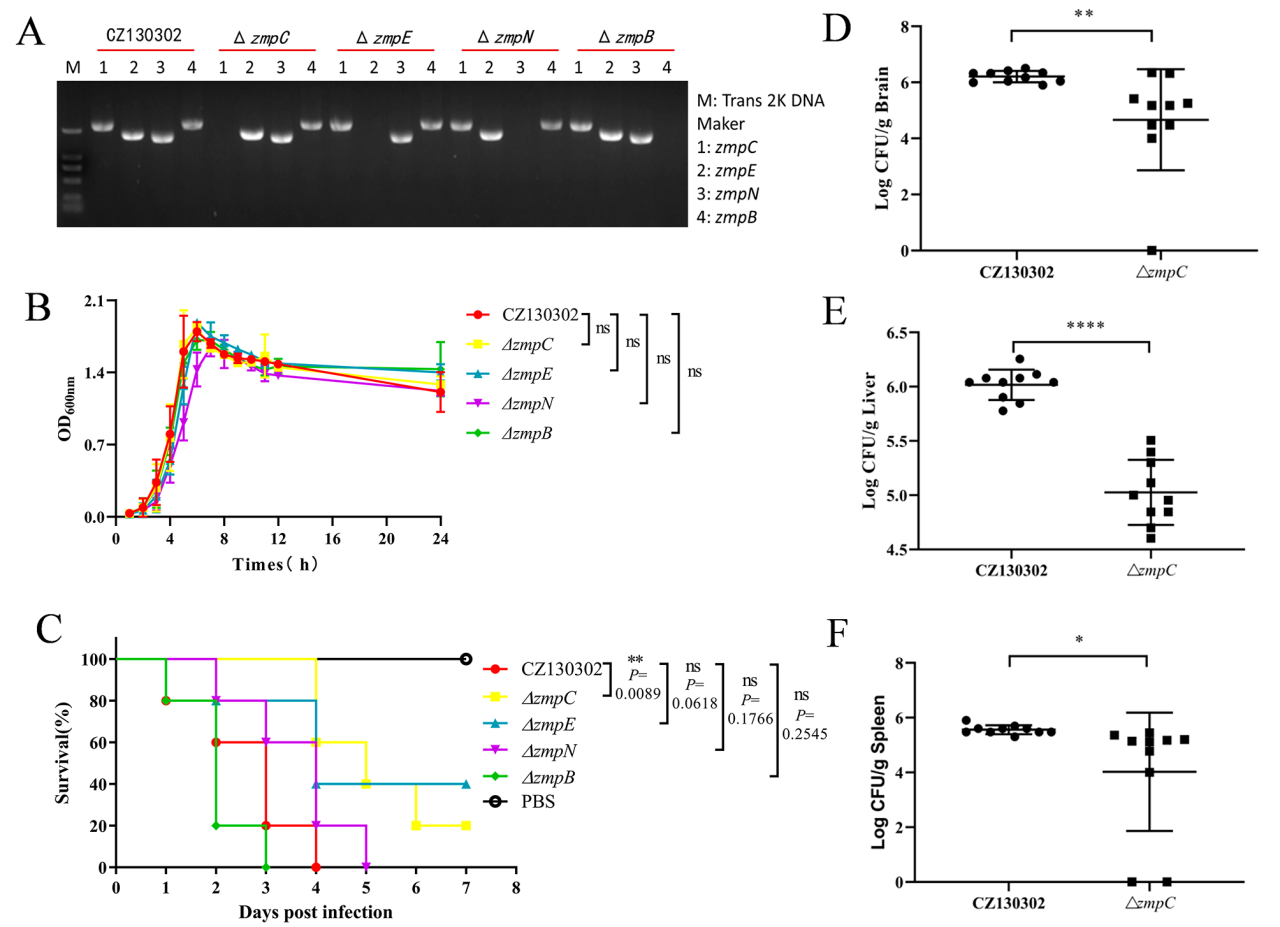
ZmpC contributes to the virulence of CZ130302. (**A**) Zmps in CZ130302 and Δ*zmp* were detected by PCR using primer pairs. The original gel is presented in Supplementary material 4. (**B**) Effect of the deletion mutants on the growth of *S. suis* CZ130302. The results are indicated as the means ± SEM of the results from 3 independent experiments (*P*>0.05). (**C**) Survival curves of 5-week-old BALB/c mice infected with wild-type or mutant strains at 5 × 10^7^ CFU/mouse. The control group received only 1×PBS. Ten mice from each group were monitored over a 7-day period. Log-rank (Mantel-Cox) test to determine differences in survival between groups: ** *P* < 0.01. Infected mice were euthanized 12 h after infection to determine the bacteria burden in the brain (**D**), liver (**E**), and spleen (**F**). Unpaired two-tailed Student’s t-test: * *P* < 0.05; ** *P* < 0.01; **** *P* < 0.0001

To explore the biological role of Zmp in *S. suis* CZ130302, we constructed a single gene deletion strain of zmp in the wild-type strain background (Fig. 2A). Next, we analyzed the effect of Zmp on the growth of CZ130302, and found that single deletion of *zmpB*, *zmpC*, *zmpN*, and *zmpE* did not affect the growth of the strain (Fig. 2B). Furthermore, CFU assay also revealed that the deletion of zmps had no effect on growth (Supplementary material 2). Since ZmpC of *S. pneumoniae* is required for virulence [17], we then performed a mouse infection assay to explore the role of Zmp in the virulence of CZ130302. The result showed that the mice infected with Δ*zmpB*, Δ*zmpE*, and Δ*zmpN* developed the same typical symptoms of meningitis (coma, drowsiness, ataxia, and walking in circles) as mice infected with CZ130302 [28], and the survival rate of mice had no significant change compared with mice infected with the wild strain (Fig. 2C). However, the survival rate of mice infected with Δ*zmpC* significantly increased, and the symptoms of meningitis in mice significantly reduced. To exclude the possibility of polarity effects, we assessed the transcript levels of upstream and downstream genes of *zmpC*. As shown in Supplementary material 3, the transcription levels of upstream and downstream genes in Δ*zmpC* did not change significantly compared with the wild-type strain. Furthermore, we tested the effect of ZmpC on the colonization of CZ130302 in vivo using the mouse infection model. Results indicated that the bacterial loads in the organs (brains, livers and spleens) of mice infected with CZ130302 were significantly higher than those of mice infected with Δ*zmpC* (Fig. 2D-F). These data suggest that ZmpC contributes to the virulence of CZ130302 and exerts certain function in the development of meningitis.

### ZmpC contributes to the development of meningitis

**Fig. 3:**
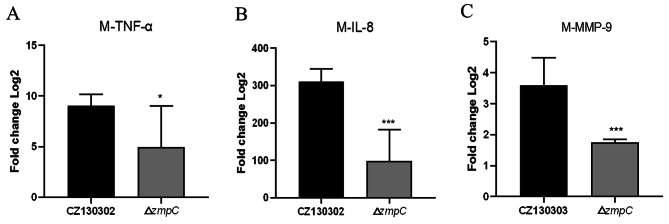
ZmpC in CZ130302 contributes to the development of meningitis. (**A**) Transcriptional level of TNF-α (**A**), IL-8 (**B**), and MMP-9 (**C**) encoding gene in the brains of the mice infected with the indicated strains. Data are represented as mean ± SEM of three independent repeats. Unpaired two-tailed Student’s t-test: * *P* < 0.05; *** *P* < 0.001

Activation of the inflammatory response is a common feature of bacterial meningitis, and interleukin-8 (IL-8) can be used as a general marker to assess the level of inflammation. A pivotal factor in the meningeal inflammatory process is tumor necrosis factor alpha (TNF-α), which can strongly stimulate the release and activation of MMPs in brain tissue [29]. MMP-9 can lyse the subendothelial basement membrane that forms the BBB around cerebral capillaries, thereby contributing to the development of brain injury [30]. To further explore the role of ZmpC in the development of meningitis, we determined the transcript levels of TNF-α, IL-8, and MMP-9 in the brain tissue of mice infected with CZ130302 and Δ*zmpC*. The results indicated that the transcript levels of TNF-α, IL-8, and MMP-9 in the brain tissues of mice infected with Δ*zmpC* were significantly lower than those of the wild-type strain at 12 h post-infection (Fig. 3). This suggests that ZmpC plays a role in the development of meningitis in the mouse model.

### ZmpC in CZ130302 can cleave human MMP-9

**Fig. 4:**
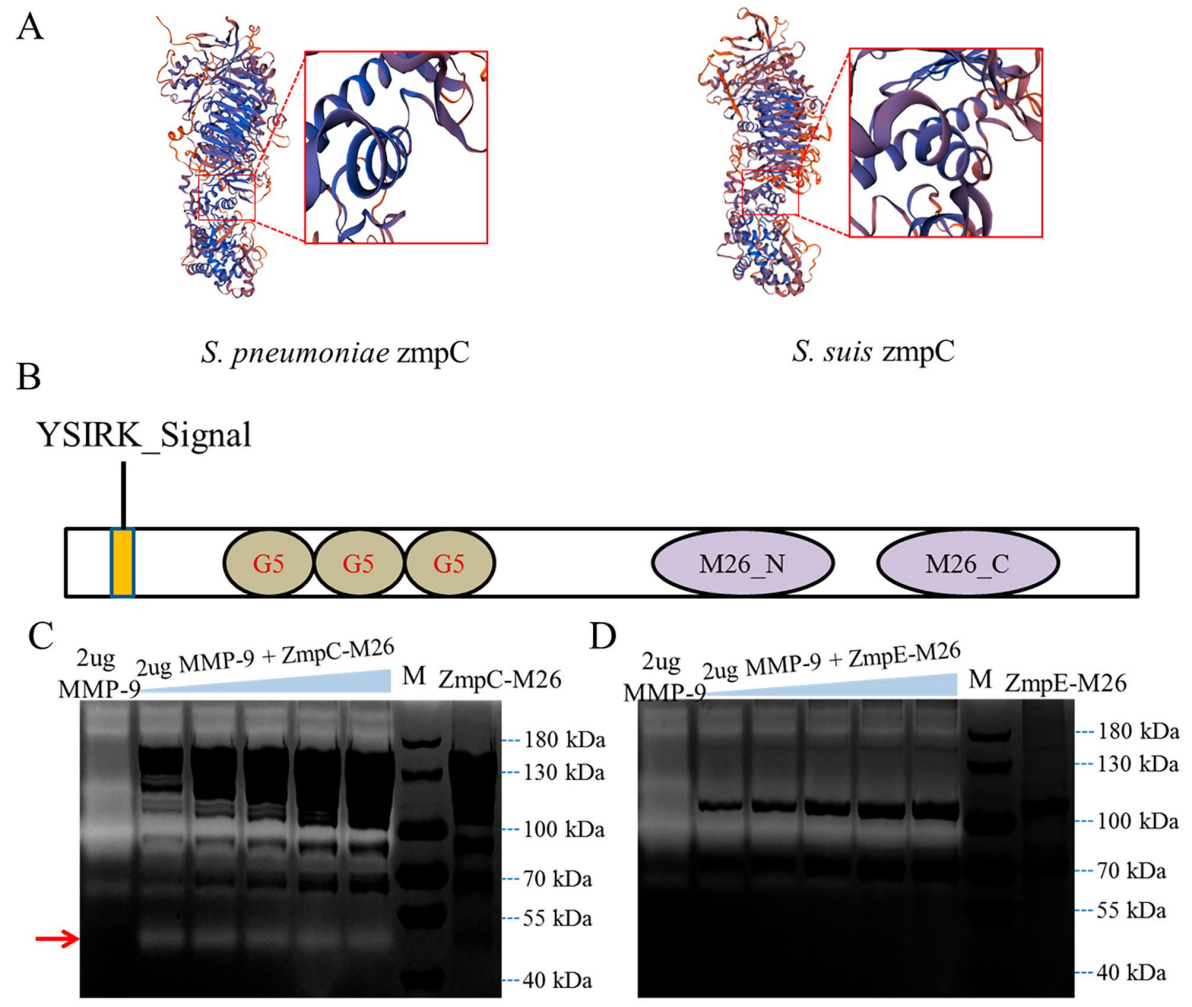
ZmpC in CZ130302 cleaves human MMP-9. (**A**) The three-dimensional structures of ZmpC in *S. pneumoniae* TIGR4 (left) and *S. suis* CZ130302 (right) were predicted using the online site SWISS-MODEL. Conserved HEXXH…E motifs in α-helix are shown in red boxes. (**B**) Schematic representation of the structure of ZmpC in *S. suis* CZ130302. ZmpC in CZ130302 contains complete M26 N-terminal and C-terminal domains. (**C**) The purified recombinant protein ZmpC-M26 was incubated with human MMP-9 for gelatin zymography analysis. The first lane indicates that the sample has only 2 µg human MMP-9, the second to sixth lanes indicate that 2 µg human MMP-9 was incubated with different concentrations of ZmpC-M26 (900 ng to 1300 ng), and the eighth lane indicates that the sample has only ZmpC-M26. The red arrow indicates the detection of a bright band of approximately 52 kDa. The results showed that ZmpC cleaves human MMP-9. (**D**) The purified recombinant protein ZmpE-M26 (900 ng to 1300 ng) was incubated with human MMP-9 for gelatin zymography analysis. No other bright bands were detected in the incubation group, indicating that ZmpE cannot cleave human MMP-9. The original gel is presented in Supplementary material 4

Previous studies have shown that ZmpC in *S. pneumoniae* can cleave human MMP-9 [18]. The previous phylogenetic analysis showed that ZmpC in CZ130302 is closely related to ZmpC (H020_RS0100375) in *S. pneumoniae* TIGR4 (Fig. 1B), suggesting that ZmpC in CZ130302 may be functionally similar to ZmpC in TIGR4. Next, we further predicted the three-dimensional structure of ZmpC in *S. pneumoniae* TIGR4 and CZ130302 using SWISS-MODEL (https://swissmodel.expasy.org). The results show that the three-dimensional structure of ZmpC in TIGR4 is highly similar to that of ZmpC in CZ130302, and the conserved HE*XX*H…E motifs are located on the α-helix (red box) (Fig. 4A). Furthermore, our protein structural analysis of ZmpC in CZ130302 found that ZmpC has YSIRK_Signal, G5 (named after its conserved glycine residue), and M26 (contains proteolytic activity) domains (Fig. 4B). To explore whether ZmpC can cleave human MMP-9, we expressed and purified ZmpC-M26 (M26 domain of ZmpC protein) for gelatin zymography analysis. As Fig. 4C shows, a bright band at approximately 52 kDa can be detected after incubation of ZmpC-M26 with MMP-9, indicating that ZmpC can cleave human MMP-9. However, no band could be detected at 52 kDa after incubation of ZmpE-M26 with MMP-9, indicating that ZmpE cannot cleave MMP-9 (Fig. 4D). These data suggest that cleavage of MMP-9 by ZmpC is specific and the cleavage site is different from that reported in previous studies [18]. Cleavage of MMP-9 by ZmpC in *S. suis* Chz type strain CZ130302 may play an important role in the development of meningitis.

### ZmpC is a critical protective antigen localized to the cell surface

**Fig. 5:**
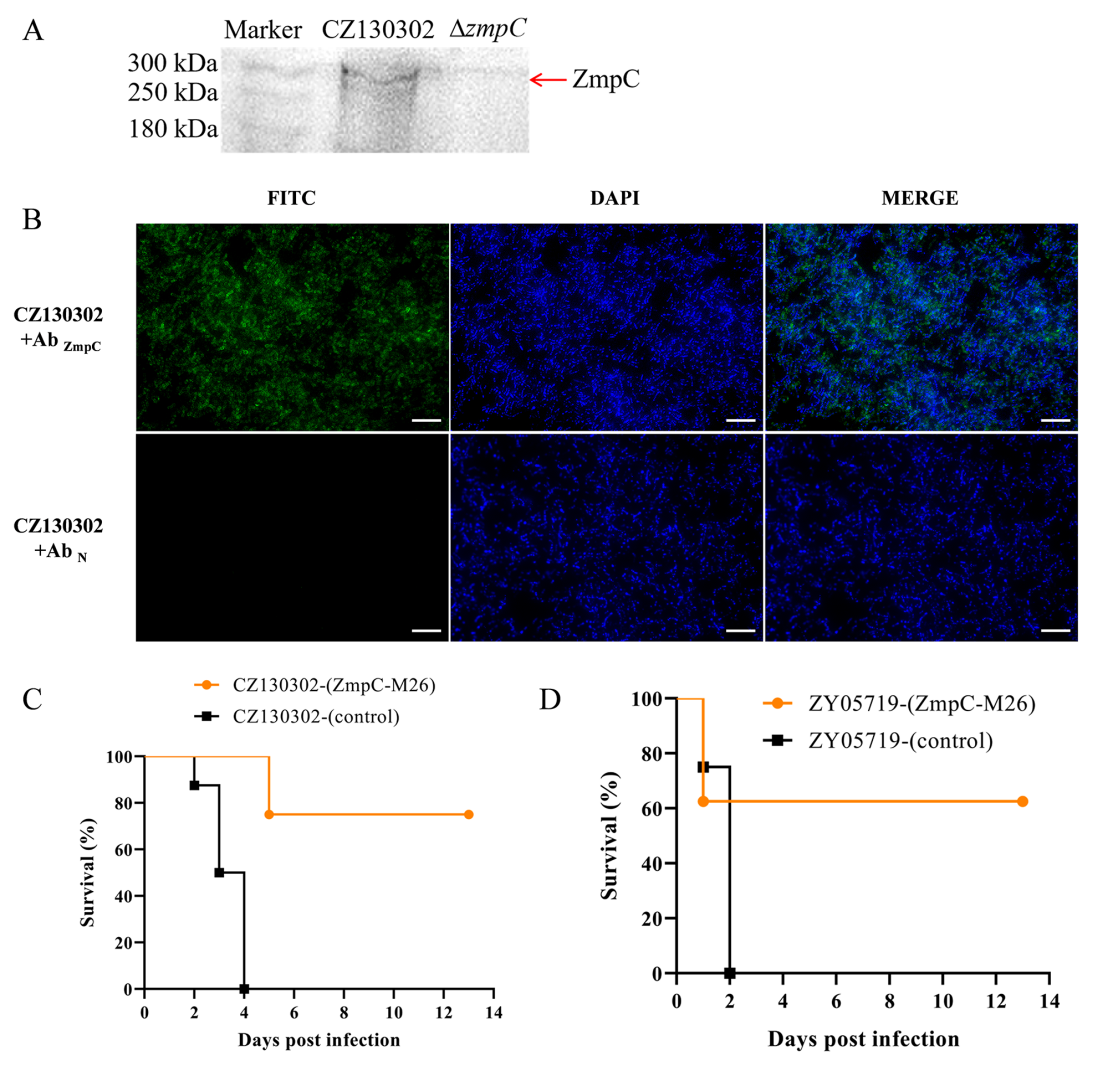
ZmpC is a protective antigen that localizes to the cell surface. (**A**) Western blot analysis identified the localization of ZmpC in CZ130302. The cell wall proteins in CZ130302 and Δ*zmpC* were extracted and Western blot was performed. A band consistent with the size of ZmpC was detected only in CZ130302 lane. The original blot is presented in Supplementary material 4. (**B**) Immunofluorescence assays were used to identify ZmpC anchored to the cell wall with anti-ZmpC-M26 and negative serum, respectively. It should be noted that intracellular proteins are not recognized by extracellular antisera. White bars represent 10 μm. To explore the protective potential of ZmpC, mice immunized with recombinant protein ZmpC-M26 were challenged with Chz type CZ130302 strain (**C**) and serotype 2 ZY05719 strain (**D**), respectively. ZmpC-M26 can provide effective protection against CZ130302 and ZY05719 infection

To determine the localization of ZmpC in CZ130302, we prepared a highly specific polyclonal antibody against ZmpC using ZmpC-M26 protein, and performed Western blot analysis. Western blot analysis of the cell wall proteins showed that a band consistent with the size of ZmpC was detected in the wild strain group, but not in the Δ*zmpC* group (Fig. 5A). Indirect immunofluorescence analysis was performed with anti-ZmpC-M26 serum and negative serum at the same time. The results showed that specific green fluorescence was detected on the cell surfaces of CZ130302 in the anti-ZmpC-M26 serum group, but not in the negative serum group (Fig. 5B). These data suggest that ZmpC in CZ130302 localizes on the bacterial cell surface.

Since ZmpC is located on the cell surface, we determined the immunogenicity and protective potential of recombinant protein ZmpC-M26 in BALB/c mice. We measured the antibody response after the fourth week of vaccinating mice with ZmpC-M26 and found that the antibody titer reached 5 × 10^5^ (data not shown), indicating that ZmpC induced a strong antibody binding response. To determine the protective potential, 24 days after vaccinating mice with ZmpC-M26, we challenged mice with CZ130302 (Chz type) and ZY05719 (serotype 2) respectively, and observed the symptoms and survival of mice daily. The results show that ZmpC-M26 can provide effective protection against CZ130302 (Chz type) and ZY05719 (serotype 2) strains, which significantly improved the survival rate of mice and alleviated the symptoms of meningitis in mice (Fig. 5C and D). Taken together, ZmpC is an important protective antigen against CZ130302 (Chz type) infection.

## Discussion

As an emerging zoonotic pathogen, *S. suis* not only causes huge economic losses to the swine industry but also causes public health problems [31]. To survive in the host, *S. suis* has evolved multiple mechanisms to counteract the host immune system, including the production of various virulence factors. The capsular polysaccharide, a crucial virulence marker, has been extensively studied in *S. suis* [32–35]. In addition, some important virulence factors such as MRP, EF, and SLY have been identified [8, 9]. Although more and more virulence factors have been characterized, the pathogenic molecular mechanism of *S. suis* still needs further exploration. Although Zmps have been identified as required for virulence of *S. pneumoniae*, the biological roles of Zmps in *S. suis* remain poorly understood. Previous study have found that ZmpC of *S. suis* serotype 2 P1/7 strain could not cleave IgA1 and PSGL-1, and could not activate MMP-9, and the inactivation of ZmpC did not affect the virulence [36]. However, the IgA1 protease in *S. suis* serotype 2 05ZYS strain can cleave human IgA and promote the virulence of 05ZYS [37]. These studies suggest that the biological roles of different Zmps in *S. suis* may be different. In the present study, our data showed that the deletion of ZmpC in the Chz type CZ130302 strain resulted in a significant attenuation of virulence in a mouse model. These results provide evidence for the role of Zmp in *S. suis* CZ130302 virulence in the mouse model.

*S. suis* is also the main pathogen causing human meningitis in Vietnam, and it is particularly urgent to study the mechanism of meningitis [38]. The *S. suis* Chz type CZ130302 strain has the ability to cause classic bacterial meningitis in piglets and mice, making it a pattern strain for studying the mechanism of meningitis [28]. Investigating the mechanism by which CZ130302 causes meningitis will help us to understand the molecular mechanism of bacterial meningitis and provide a theoretical basis for the prevention and control of bacterial meningitis disease. Previous studies have identified a genomic island (50 K GI) that encodes the SecY2/A2 secretion system and a secreted protein, SssP1, on the CZ130302 genome [39]. The N-terminus of SssP1, which is secreted by the SecY2/A2 system, contains a specific KXYKXGKXW signal peptide and a serine-rich repeat adhesion glycoprotein AST domain. Deletion of either SecY2/A2 or SssP1 led to a significant decrease in bacterial virulence. In addition, SssP1 plays a key facilitating role in the development of meningitis [40]. SssP1 promotes CZ130302 adhesion to and invasion into host cells through interaction with vimentin, and the salivation of vimentin is necessary for the binding of SssP1 to vimentin. However, the mechanism of CZ130302 penetrating the BBB remains to be studied. Matrix metalloproteinases (MMPs) are a family of Zn^+^-dependent endopeptidases that degrade the subendothelial basement membrane, which forms the BBB around cerebral capillaries [41–43]. MMP-9 acts as a 92 kDa type IV collagenase (gelatinase B) that specifically degrades type IV collagen, which is a crucial structural component of the perivascular basement membrane [44]. MMP-9 has been shown to induce BBB disruption and promote leukocyte extravasation in experimental bacterial meningitis and other models of neuroinflammation [21, 42, 43, 45–47]. In this study, we demonstrated that ZmpC in CZ130302 can cleave human MMP-9, which is consistent with *S. pneumoniae* ZmpC [18]. These results suggest that ZmpC may also cleave porcine MMP-9, but further validation is required. The expression, secretion, and activity levels of MMPs are tightly regulated [48, 49]. All MMPs have a pro-domain that remains enzymatically inactive until protease activity is required [50]. The pro-domain serves as an internal inhibitor of MMP activity, and activation occurs when cleavage of the pro-domain leads to a conformational change into the active form [50]. This activation model has been confirmed in the resolution of the crystal structure of the MMP-1 catalytic domain [51]. In addition, cleavage of MMP-9 may substantially affect MMP-9 activity by either activating the pro-protease or cleaving the inhibitor binding domain [50, 52]. In this study, ZmpC-mediated cleavage in *S. suis* CZ130302 may also activate MMP-9, thereby promoting the development of meningitis. Furthermore, our data indicated that ZmpC significantly promoted the expression of MMP-9 in mouse brain tissue. In conclusion, ZmpC in CZ130302 plays an important role in damaging the BBB, which will provide a new perspective for studying the mechanism of BBB impairment.

With indiscriminate use of antibiotics causing the emergence of ever more antibiotic resistant pathogens, vaccine research is particularly important. At present, inactivated vaccines of *S. suis* serotypes 2 and 9 have been developed. These inactivated vaccines have the advantages of short preparation cycle, safety, and easy storage. However, these inactivated vaccines often require large dose for immunization and are only protective against infection with the same serotype of *S. suis*. In addition, study has reported a subunit vaccine composed of MRP and EF, which confers significant protection from *S. suis* serotype 2 infection [53]. There are few studies on vaccines against multiple serotypes, and our study found that ZmpC can be used to effectively resist *S. suis* serotype 2 and Chz type strains infections. These data provide a rationale for developing *S. suis* vaccine.

In summary, inactivation of ZmpC significantly attenuated the virulence of CZ130302 in a mouse model, suggesting that ZmpC might be part of the *S. suis* Chz type strains virulence factor arsenal. Moreover, ZmpC promotes the expression of inflammatory factors and cleaves human MMP-9, suggesting that ZmpC may be a key factor in the meningitis caused by *S. suis* Chz type CZ130302. The characterization of ZmpC provides several important insights for both research on pathogenic mechanisms and vaccine development in *S. suis* CZ130302.

### Electronic supplementary material

Below is the link to the electronic supplementary material.

Supplementary Material 1

Supplementary Material 2

Supplementary Material 3

Supplementary Material 4

## Data Availability

All data and materials are available at OIE Reference Lab for Swine Streptococcosis, College of Veterinary Medicine, Nanjing Agricultural University, Nanjing 210095, China.
