# Supplementary material for: Insight into the role of Streptococcus suis zinc metalloprotease C from the new serotype causing meningitis in piglets
Source: BMC Vet Res. 2024 Jul 30;20:337. doi: 10.1186/s12917-024-03893-4 (PMC11290213; doi:10.1186/s12917-024-03893-4)
Supplement: Supplementary file 1 — Supplementary Material 1 [file 12917_2024_3893_MOESM1_ESM.docx]

**Supplementary material 1**

**All primers used in this study.** All primers were designed using the Primer Premier Software (version: 5.0) and synthesized by Shanghai Sangon Biotech Co., Ltd., China.

| Primer | Sequence (5’—3’) ^a^ | Comment |
| --- | --- | --- |
| Primer for construction of deletion strains | |  |
| zmpC-S-F | GCGAACCTTTATCACTACCCT | Upstream of the fusion fragment for  Δ*zmpC* (Step 1) |
| zmpC-S-R | TCAGCATTATCCATCTATGGTGAATGCCCTATT |  |
| zmpC-X-F | GGTAATCAGATTACAAAGAGCGGATAGATCAAT | Downstream of the fusion fragment  for Δ*zmpC* (Step 1) |
| zmpC-X-R | GGCGACTCCTAAGAAGACAGA |  |
| zmpC-RH-F | AATGGGCGTCAAGGTCAAGTA | The fusion fragment of three for  Δ*zmpC* (Step 1) |
| zmpC-RH-R | CTATTGCGACCGCCTGT |  |
| zmpC-U-F | ATCTACGGTTTGGGATCGC | Upstream of the fusion fragment for  Δ*zmpC* (Step 2) |
| zmpC-U-R | ATCCGCTCTTTGATCTATGGTGAATGCCCTATT |  |
| zmpC-D-F | TTCACCATAGATCAAAGAGCGGATAGATCAA | Downstream of the fusion fragment  for Δ*zmpC* (Step 2) |
| zmpC-D-R | CCTATAAACGCAAGAAAGCTA |  |
| zmpE-S-F | TGCTGCTGGTATCAAACAGGC | Upstream of the fusion fragment for  Δ*zmpE* (Step 1) |
| zmpE-S-R | TCAGCATTATCCTTTCCAGATGAAAAAACCTCG |  |
| zmpE-X-F | GGTAATCAGATTATATTTTCGGTTTCTTCCTT | Downstream of the fusion fragment  for Δ*zmpE*(Step 1) |
| zmpE-X-R | CAGCCTTCTGAGGTGCATTGG |  |
| zmpE-RH-F | GATTAACATCATCTTGGACGG | The fusion fragment of three for  Δ*zmpE* (Step 1) |
| zmpE-RH-R | ATTTGATACTGCCCTTCTGTT |  |
| zmpE-U-F | TTGGCAGCCCTTGCTGAAT | Upstream of the fusion fragment for  Δ*zmpE*(Step 2) |
| zmpE-U-R | AACCGAAAATATTTTCCAGATGAAAAAACCTCG |  |
| zmpE-D-F | TTCATCTGGAAAATATTTTCGGTTTCTTCCTT | Downstream of the fusion fragment  for Δ*zmpE* (Step 2) |
| zmpE-D-R | CCACCCAGACATGAGCGTCAA |  |
| zmpN-S-F | GCCCACTCTGGATAAATCGC | Upstream of the fusion fragment for  Δ*zmpN* (Step 1) |
| zmpN-S-R | TCAGCATTATCCACAGCCGTTCGTTCGCTCA |  |
| zmpN-X-F | GGTAATCAGATTCGCTGACAAATTTATCGCAAC | Downstream of the fusion fragment for Δ*zmpN*(Step 1) |
| zmpN-X-R | TGCCGGTGCTTATGACCTATT |  |
| zmpN-RH-F | ATCGCAAGGTCAGAGTATGAG | The fusion fragment of three for Δ*zmpN* (Step 1) |
| zmpN-RH-R | ATGCCTTTCAGCCCTTGTCTA |  |
| zmpN-U-F | TCTTATCCAAGCCCTGAACA | Upstream of the fusion fragment for  Δ*zmpN* (Step 2) |
| zmpN-U-R | AATTTGTCAGCGACAGCCGTTCGTTCGCTCA |  |
| zmpN-D-F | ACGAACGGCTGTCGCTGACAAATTTATCGCAAC | Downstream of the fusion fragment  for Δ*zmpN* (Step 2) |
| zmpN-D-R | GGCTTGACAGGCTTTTCCGTG |  |
| zmpB-S-F | TAGGCCAGCGGTGCAATCTCG | Upstream of the fusion fragment for Δ*zmpB* (Step 1) |
| zmpB-S-R | TCAGCATTATCCCCCTCCGCAAGGATATGTTTA |  |
| zmpB-X-F | GGTAATCAGATTCATCAATGCCCCAAATACTCT | Downstream of the fusion fragment  for Δ*zmpB* (Step 1) |
| zmpB-X-R | AGGGGATTTTCAAAGAATTTA |  |
| zmpB-RH-F | GCCAGCGGTGCAATCTCGATA | The fusion fragment of three for Δ*zmpB* (Step 1) |
| zmpB-RH-R | CTTCTAAGAAGGCTTCCAATC |  |
| zmpB-U-F | CCTTGGCTGAAAAATGCACAA | Upstream of the fusion fragment for  Δ*zmpB* (Step 2) |
| zmpB-U-R | GGGGCATTGATGCCCTCCGCAAGGATATGTTTA |  |
| zmpB-D-F | CCTTGCGGAGGGCATCAATGCCCCAAATACTCT | Downstream of the fusion fragment for Δ*zmpB*(Step 2) |
| zmpB-D-R | GAAAACGGCTTGAAAGTGACT |  |
| Primer for deletion detection | | |
| zmpC-F | ATGCCGTTCGATATTGTGGTC | Primers for detection of *zmpC* |
| zmpC-R | TTCCGCTTTGCCAACCTGT |  |
| zmpE-F | ATGTGGAAGAAGAGCTCGG | Primers for detection of *zmpE* |
| zmpE-R | TCGATAAGGCTTGTTTCCAGA |  |
| zmpN-F | TGCCCGTCTTGCATACATAG | Primers for detection of *zmpN* |
| zmpN-R | TTTCAGCCAAAGACCTCATTT |  |
| zmpB-F | CTGGGGTAGTGGATAGAGACG | Primers for detection of *zmpB* |
| zmpB-R | TCAGAAACGGAAAAAAATGAT |  |
| sacB+spc-JC-F | TTACGGCAAACAAACACTGAC | Primers for detection of *sacB* and *spc* |
| sacB+spc-JC-R | TTTGGGAGGATGATTCCACGG |  |
| Primers for Q-PCR to detect the expression levels | | |
| CVO91_05665-F | GATGAAGCCTCTGGAATGTATGA | Primers for detecting the expression levels of the *CVO91_05665* |
| CVO91_05665-R | AAGTCACGTCCTTGTTGGATAG |  |
| CVO91_05670-F | GACTTGACCGACTACCTGAAAG | Primers for detecting the expression levels of the *CVO91_05670* |
| CVO91_05670-R | CAAAGACACCTAAGCGCAAATC |  |
| CVO91_05675-F | GGTCCCAAGTGCTGATCTTTAT | Primers for detecting the expression levels of the *CVO91_05675* |
| CVO91_05675-R | GCGGACACCCTTGATAGTTT |  |
| CVO91_05680-F | CCCATCAGTCGCAAGGAAATA | Primers for detecting the expression levels of the *CVO91_05680* |
| CVO91_05680-R | GAGAATAGGCTGCTCCAACAA |  |
| CVO91_05685-F | CCATTGAAATCATGACCAACGAG | Primers for detecting the expression levels of the *CVO91_05685* |
| CVO91_05685-R | CGGGTTGATACACAACCTTCT |  |
| GAPDH-F | GTTTGGCACCAATGGCTAAAG | Design based on the reference [1] |
| GAPDH-R | CCAGTGTAAGCGTGGATTGT |  |
| M-TNF-α-F | AAGCCTGTAGCCCACGTCGTAA | Primers for detecting the expression levels of the TNF-α |
| M-TNF-α-R | GGCACCACTAGTTGGTTGTCTTTG |  |
| M-IL-8-F | AGGGCGGTCAAAAAGTTTGC | Primers for detecting the expression levels of the IL-8 |
| M-IL-8-R | CAGGTACGATCCAGGCTTCC |  |
| M-MMP-9-F | TTGAGTCCGGCAGACAATCC | Primers for detecting the expression levels of the MMP-9 |
| M-MMP-9-R | ACTTCCAGTACCAACCGTCC |  |
| β-Actin-F | CTTCCAGCCTTCCTTCCTGG | Design based on the reference [2] |
| β-Actin-R | CTGTGTTGGCGTACAGGTCT |  |
| Primer for construction of expression vectors | | |
| ZmpC-M26-F | CAAATGGGTCGCggatccGTAGAAAAAGAGGGGAAAGAC | Primers for construction  of ZmpC-M26 |
| ZmpC-M26-R | GTGGTGGTGGTGctcgagTGTTCTTAACATAAACGTCTATGG |  |
| ZmpE-M26-F | CAAATGGGTCGCggatccCAAAATGCTCAGGCTGAACGA | Primers for construction  of ZmpE-M26 |
| ZmpE-M26-R | GTGGTGGTGGTGctcgagCTTCCATCATTCTCCAAATATAGA |  |
| Primers for detecting the vectors | | |
| T7-F | TAATACGACTCACTATAGGG | Primers for detecting the  vectors |
| T7-R | GCTAGTTATTGCTCAGCGG |  |

^a^Underlined nucleotides denote reverse complement; Lowercase nucleotides denote restriction enzyme sites.

1. Ju CX, Gu HW, Lu CP. Characterization and functional analysis of atl, a novel gene encoding autolysin in Streptococcus suis. J Bacteriol. 2012 Mar;194(6):1464-73.
2. Liu J, Zhong X, He Z, Zhang J, Bai J, Liu G, Liang Y, Ya L, Qin X. Erythromycin Suppresses the Cigarette Smoke Extract-Exposed Dendritic Cell-Mediated Polarization of CD4^+^ T Cells into Th17 Cells. J Immunol Res. 2020 Jan 21;2020:1387952.
