# Supplementary material for: Insight into the role of Streptococcus suis zinc metalloprotease C from the new serotype causing meningitis in piglets
Source: BMC Vet Res. 2024 Jul 30;20:337. doi: 10.1186/s12917-024-03893-4 (PMC11290213; doi:10.1186/s12917-024-03893-4)

**Supplementary material 2**

**CFU assay of zmps deletion strains.**

The number of viable bacteria in the deletion strains was determined by counting CFU. The values of CFU per milliliter represented the mean ± SD of three biological repeats.


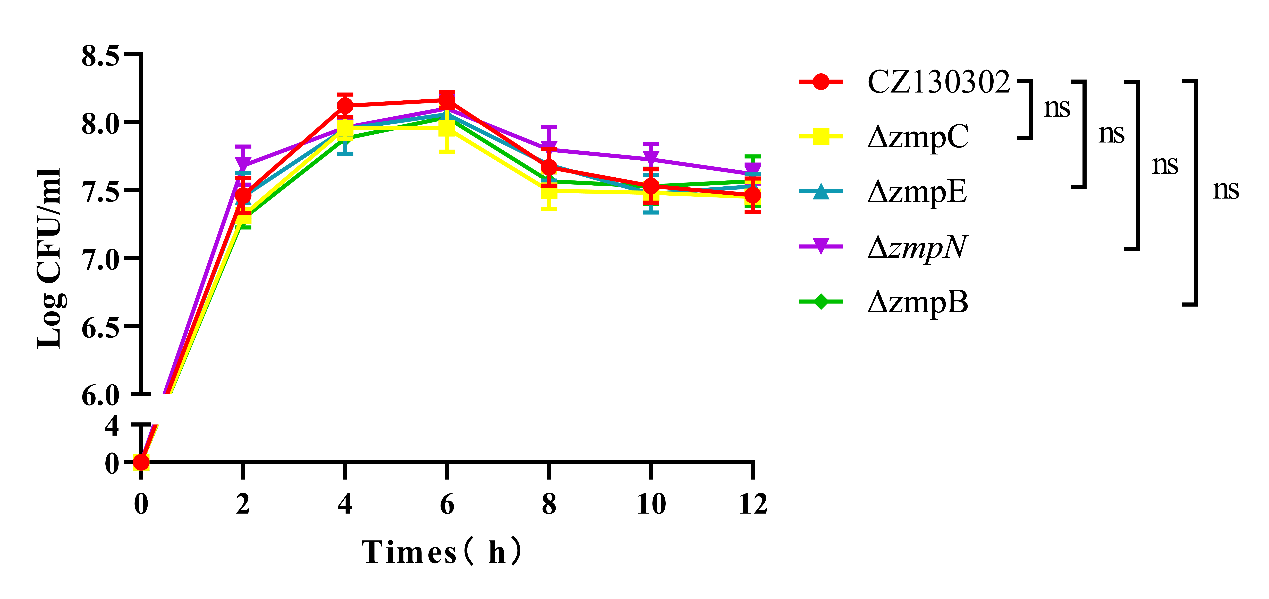

Supplement: Supplementary file 2 — Supplementary Material 2 [file 12917_2024_3893_MOESM2_ESM.docx]
