# Supplementary material for: Insight into the role of Streptococcus suis zinc metalloprotease C from the new serotype causing meningitis in piglets
Source: BMC Vet Res. 2024 Jul 30;20:337. doi: 10.1186/s12917-024-03893-4 (PMC11290213; doi:10.1186/s12917-024-03893-4)
Supplement: Supplementary file 3 — Supplementary Material 3 [file 12917_2024_3893_MOESM3_ESM.docx]

**Supplementary material 3**

**The effect of zmpC deletion on the transcription level of its flank genes.** Expression levels of upstream and downstream genes in Δ*zmpC* strain as measured by qRT-PCR. The relative expression levels represented the mean ± SD of three biological repeats.

**
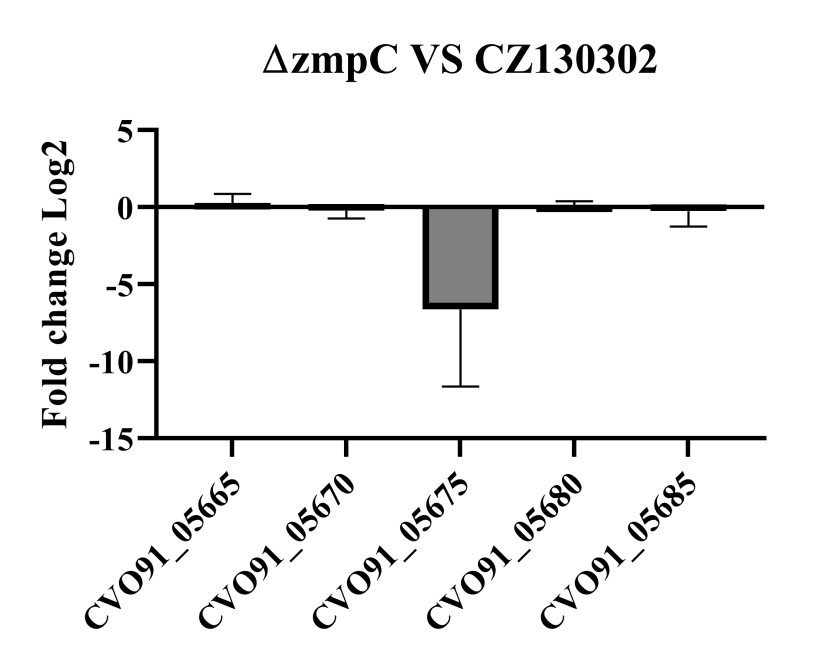
**
