# Supplementary material for: Insight into the role of Streptococcus suis zinc metalloprotease C from the new serotype causing meningitis in piglets
Source: BMC Vet Res. 2024 Jul 30;20:337. doi: 10.1186/s12917-024-03893-4 (PMC11290213; doi:10.1186/s12917-024-03893-4)
Supplement: Supplementary file 4 — Supplementary Material 4 [file 12917_2024_3893_MOESM4_ESM.docx]

**Supplementary material 4**

**The full-length gels and blots.**

**Fig. 2A** Zmps in CZ130302 and Δ*zmp* were detected by PCR using primer pairs.

**
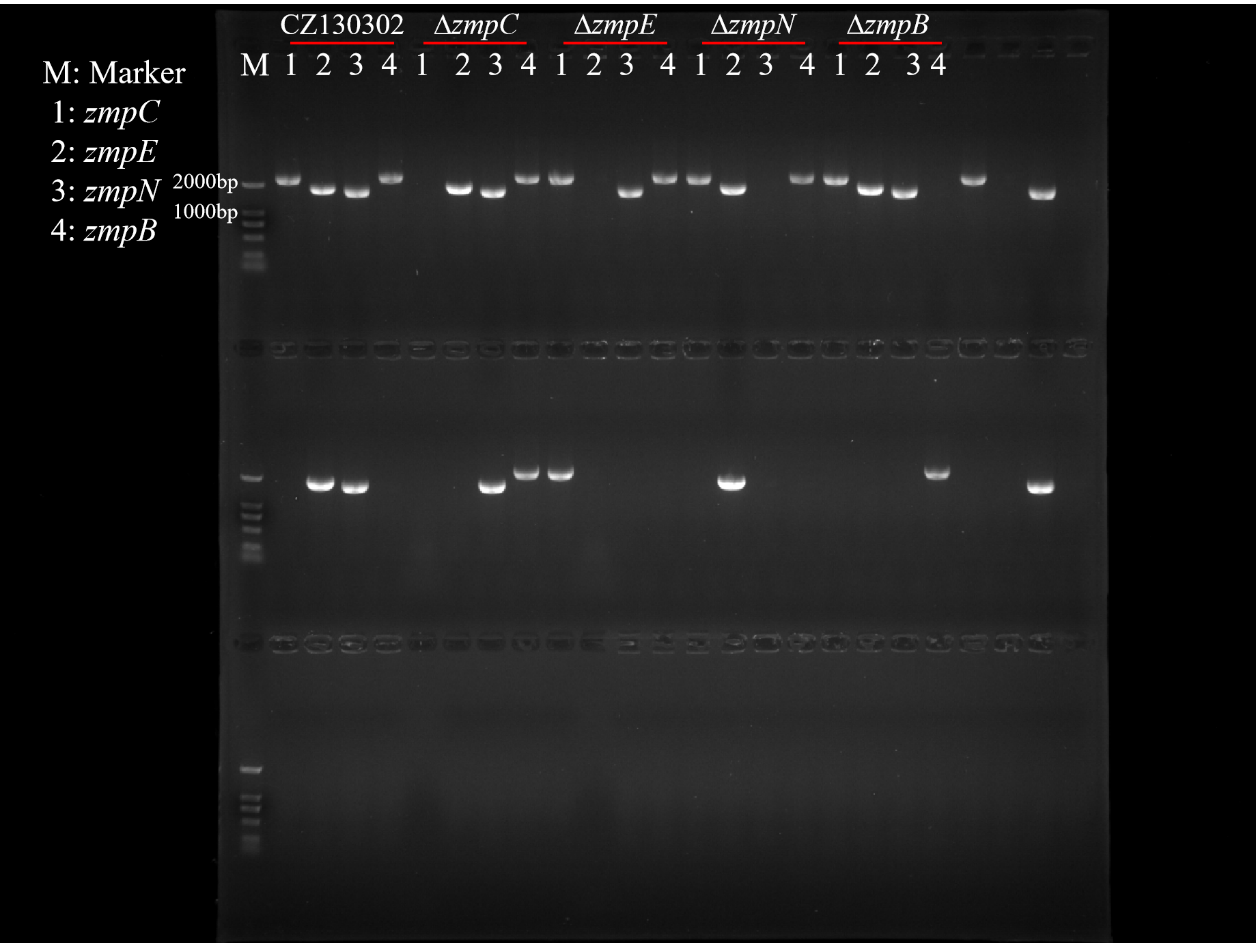
**

Fig. 4C The purified recombinant protein ZmpC-M26 was incubated with human MMP-9 for gelatin zymography analysis.

**
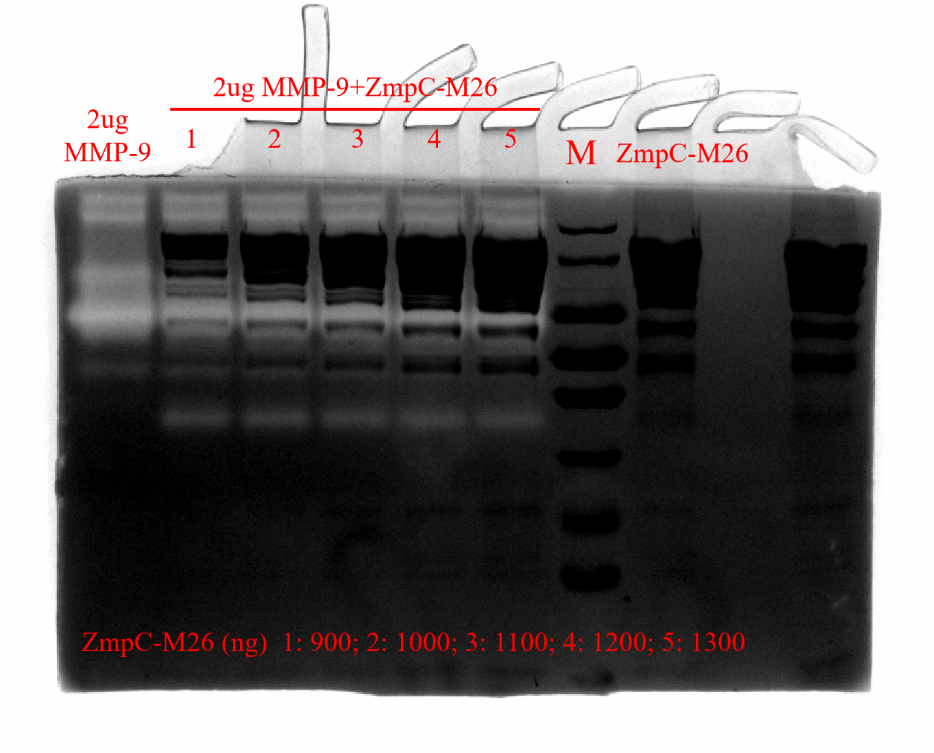
**

Fig. 4D The purified recombinant protein ZmpE-M26 was incubated with human MMP-9 for gelatin zymography analysis.


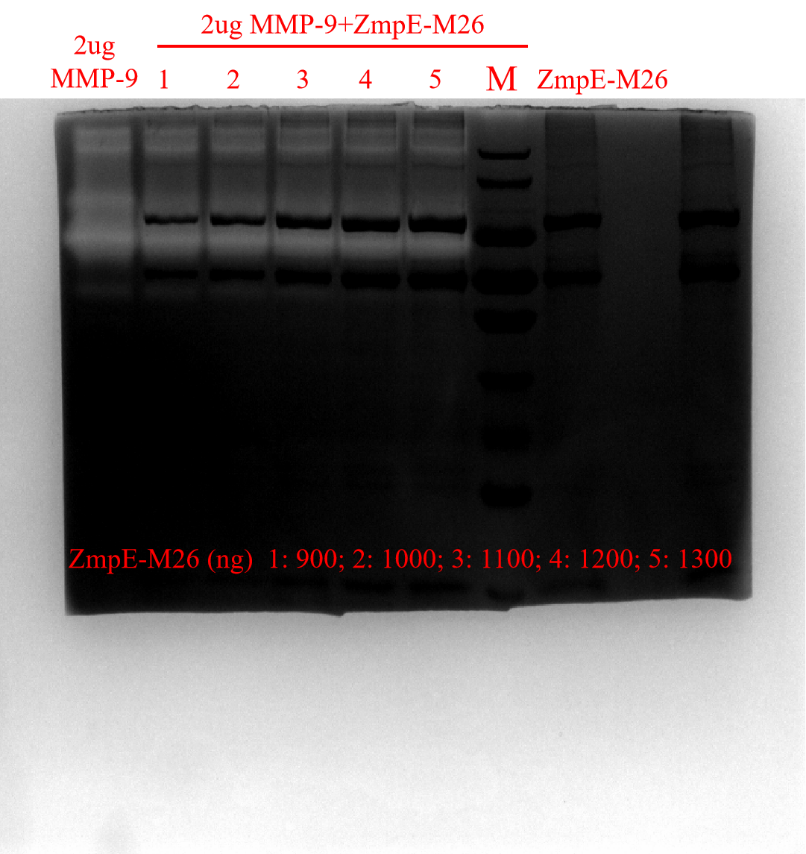


Fig. 5A Western blot analysis identified the localization of ZmpC in CZ130302.

**
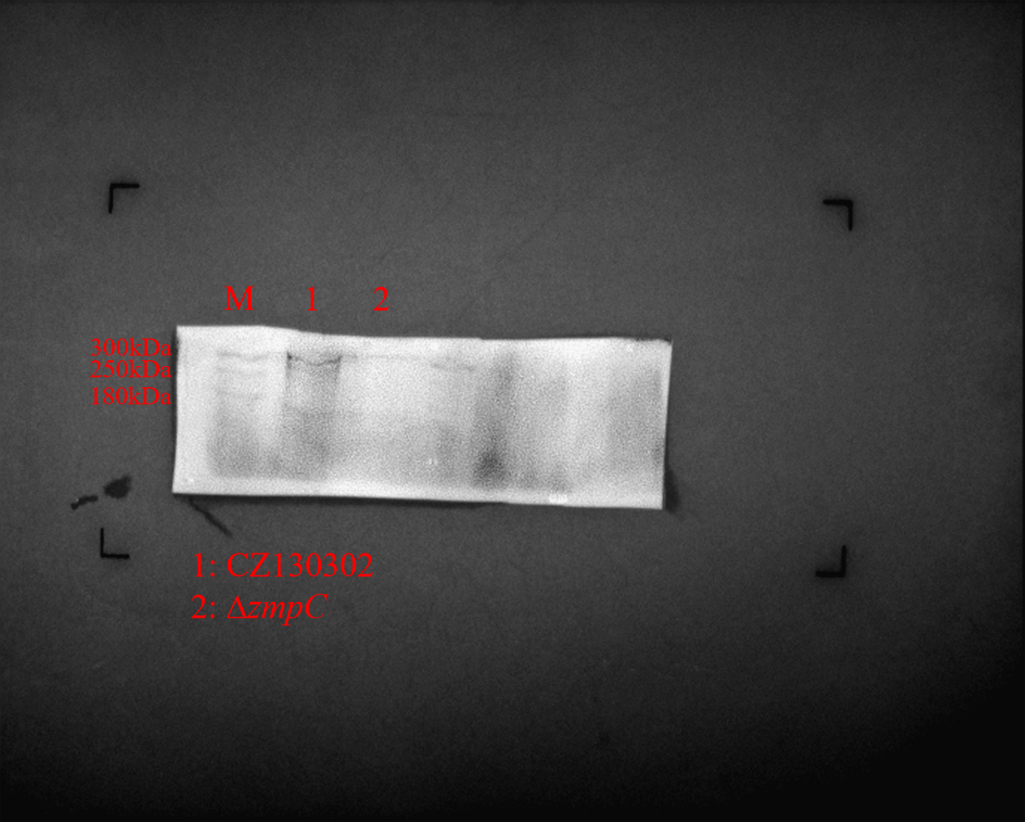
**
